# Restoring Perivascular Adipose Tissue Function in Obesity Using Exercise

**DOI:** 10.1007/s10557-020-07136-0

**Published:** 2021-03-09

**Authors:** Sophie N Saxton, Lauren K Toms, Robert G Aldous, Sarah B Withers, Jacqueline Ohanian, Anthony M Heagerty

**Affiliations:** 1 Division of Cardiovascular Sciences, University of Manchester, Manchester, UK; 2 The Lydia Becker Institute of Immunology & Inflammation, University of Manchester, Manchester, UK; 3 King’s College Hospital, London, UK; 4 School of Science, Engineering and Environment, University of Salford, Salford, UK; 5 Division of Cardiovascular Sciences, Manchester Academic Health Science Centre, Core Technology Facility (3rd floor), 46 Grafton Street, Manchester, M13 9NT UK

**Keywords:** Adipocytes, Adrenoceptors, Obesity, Exercise, Sympathetic nervous system

## Abstract

**Purpose:**

Perivascular adipose tissue (PVAT) exerts an anti-contractile effect which is vital in regulating vascular tone. This effect is mediated via sympathetic nervous stimulation of PVAT by a mechanism which involves noradrenaline uptake through organic cation transporter 3 (OCT3) and β_3_-adrenoceptor-mediated adiponectin release. In obesity, autonomic dysfunction occurs, which may result in a loss of PVAT function and subsequent vascular disease. Accordingly, we have investigated abnormalities in obese PVAT, and the potential for exercise in restoring function.

**Methods:**

Vascular contractility to electrical field stimulation (EFS) was assessed ex vivo in the presence of pharmacological tools in ±PVAT vessels from obese and exercised obese mice. Immunohistochemistry was used to detect changes in expression of β_3_-adrenoceptors, OCT3 and tumour necrosis factor-α (TNFα) in PVAT.

**Results:**

High fat feeding induced hypertension, hyperglycaemia, and hyperinsulinaemia, which was reversed using exercise, independent of weight loss. Obesity induced a loss of the PVAT anti-contractile effect, which could not be restored via β_3_-adrenoceptor activation. Moreover, adiponectin no longer exerts vasodilation. Additionally, exercise reversed PVAT dysfunction in obesity by reducing inflammation of PVAT and increasing β_3_-adrenoceptor and OCT3 expression, which were downregulated in obesity. Furthermore, the vasodilator effects of adiponectin were restored.

**Conclusion:**

Loss of neutrally mediated PVAT anti-contractile function in obesity will contribute to the development of hypertension and type II diabetes. Exercise training will restore function and treat the vascular complications of obesity.

**Supplementary Information:**

The online version contains supplementary material available at 10.1007/s10557-020-07136-0.

## Introduction

Perivascular adipose tissue (PVAT) is a highly metabolically active tissue which surrounds the majority of blood vessels and contributes to the modulation of vascular tone [1–3]. Previously, we have presented evidence that the mechanisms of this effect are mediated via the sympathetic nervous system and are two-fold [4]; first, sympathetic nerve-derived noradrenaline (NA) activates adipocyte β_3_-adrenoceptors, which triggers the release of the vasodilator adiponectin. Second, excess NA is sequestered by adipocytes via organic cation transporter 3 (OCT3). In obesity, PVAT function is lost, which may contribute to development of metabolic syndrome [5–7]. However, the mechanisms behind this loss of function are unclear.

It is well known that in obesity, pathological over-activity of the sympathetic nervous system occurs [8, 9]. Cardiac β-adrenoceptors become desensitised in heart failure, as a result of sympathetic nerve over-activity [10]. It is possible that in PVAT, increased sympathetic activity could result in a desensitisation of β_3_-adrenoceptors, leading to reduced adiponectin release. Bioavailability of adiponectin has already been demonstrated to be reduced in human obesity [11], hypertension [12], and type II diabetes [13].

In obesity, PVAT morphology changes, and adipocyte size and number increase. However, there is no increase in blood supply, leading to hypoxia and chronic inflammation [14, 15]. By subjecting human and mouse mesenteric arteries to hypoxia, the loss of the PVAT anti-contractile effect can be replicated [6, 16]. There is substantial evidence for the role of inflammation in the loss of PVAT function [17]. Hypoxia-induced loss of anti-contractile function is prevented in the absence of macrophages from the PVAT [16]. Moreover, cytokine antagonists can be used to rescue hypoxia-induced damage to PVAT function [6]. This suggests that hypoxia induces an inflammatory response involving classical macrophage activation, leading to the release of cytokines into the local environment, which damages the adipocytes. Furthermore, hypoxia-induced damage can also be rescued using free radical scavengers [6, 16, 18], illustrating that hypoxia causes oxidative stress, which can ultimately cause damage to the surrounding environment.

The role of adiponectin in the inflammatory response may contribute to insulin insensitivity. Adiponectin is an inhibitor of the cytokine tumour necrosis factor-α TNFα), which inhibits insulin signalling [19–22]. Therefore, reduced levels of adiponectin will increase TNFα-mediated inhibition of insulin signalling. Furthermore, TNFα expression is increased in obesity [22], further inhibiting the vasodilator effects of insulin in skeletal muscle, reducing blood flow and glucose uptake, and leading to type-II diabetes.

Sympathetic nerves play a role in recruiting inflammatory mediators, and a number of studies have demonstrated that exercise, which is considered to be healthy sympathetic stimulation, can reduce inflammation in obesity [23–26]. In a study of inflammation in skeletal muscle, forced interval training in high fat-fed mice significantly reduced macrophage infiltration [23]. Interestingly, these effects were independent of weight loss. In a similar study using forced exercise, macrophage infiltration and inflammatory cytokine expression were reduced in the adipocytes of epididymal fat pads of obese exercised trained mice, again independent of weight loss [25]. Another study using obese mice provided with a wheel for voluntary exercise [24] demonstrated significant improvements in insulin-sensitivity, glucose tolerance, and reduced total body fat mass and exhibited a significant reduction in inflammatory cytokine expression including TNFα. In diabetic obese human patients, exercise has been shown to reduce TNFα and interleukin-6 expression in subcutaneous adipose [27]. The results of these studies could be applied to reduce macrophage infiltration and inflammatory cytokine expression in PVAT surrounding resistance arteries, reversing obesity-induced damage to PVAT, and alleviating the vascular complications of obesity, i.e., hypertension and diabetes.

The beneficial effects of exercise on brown adipose tissue function surrounding the aorta has been well documented [28–31]; however, the effects of exercise on white mesenteric PVAT function and small resistance arteries have not yet been characterised. Previous weight loss studies such as bariatric surgery [32], and caloric restriction [33] have shown success in restoring PVAT function in obesity. In addition, pharmacological intervention to increase endothelial nitric oxide synthase (NOS) phosphorylation in obesity successfully restored function of aortic PVAT [34]. Alongside improvements in PVAT function, these studies also demonstrated improvements in insulin sensitivity and blood pressure, highlighting the importance of restoring PVAT function to treat the vascular complications of obesity.

The present study was designed to examine the mechanisms of PVAT dysfunction in obesity and the potential for a significant lifestyle intervention in restoring function. We tested the hypothesis that a loss of PVAT function in obesity can be reversed via exercise. We anticipate that in obesity, PVAT becomes inflamed, and expression of β_3_-adrenoceptors and OCT3 are reduced. These changes in the PVAT environment will be reversed by exercise. In addition, we hypothesise that the resultant symptoms of metabolic syndrome in obesity will be alleviated by exercise.

## Materials and Methods

### Animal Care and Ex Vivo Measurements

All experiments were performed in accordance with the appropriate Home Office project licence and complied with the UK Animal (Scientific Procedures) Act 1986. Male C57BL/6j mice were randomly assigned to three groups; control, obese, and exercised obese (see below) and aged until 18–20 weeks. Animals were housed under a 12-h light/12-h dark cycle and provided with food and water ad libitum. The body weights of all groups were recorded every 2 weeks from 8 weeks of age. Mice were fasted overnight and sacrificed using CO_2_ asphyxiation. Prior to sacrifice, blood pressure was recorded in conscious, restrained animals using the CODA tail cuff blood pressure monitoring system (Kent Scientific, USA). Immediately after sacrifice mixed blood samples were collected by severing the thoracic aorta. Blood glucose concentration was measured immediately using an automatic blood glucose system (Contour, Bayer Consumer Care AG, Basel, Switzerland). Following euthanisation, the mesentery was exposed and removed. Epididymal fat pads, hearts, and kidneys were collected and weighed.

### Control Mice

Control mice were fed a standard chow diet ad libitum (7.42% fat, cat no. BK001, SDS Diets, UK) until they were euthanised at 18–20 weeks old (*n* = 20).

### Obese Mice: Diet-Induced Obesity

Male C57BL/6j mice (Harlan Laboratories, UK) were purchased at 6 weeks old and allowed a 2-week acclimatisation period before being placed on a 60% kcal from fat diet (cat no. 824054, SDS Diets, UK) ad libitum for 10–12 weeks and euthanised at 18–20 weeks (*n* = 44). A 10–25% increase in body weight as compared with age-matched controls is considered as moderate obesity in rodents [35]; therefore, to eliminate mice which may be resistant to obesity, mice with less than a 10% increase in body weight as compared with healthy controls were excluded from this study.

### Exercised Obese Mice

For exercised obese mice, male C57BL/6j mice followed the diet-induced obesity protocol as described above. At week five, forced swim training commenced. Mice were initially exercised for 15 min once a day, and the duration of exercise increased in daily increments of 15 min until reaching a final duration of 60 min. From week six, mice were exercised five times per week. Mice were swum in tanks with a surface area of 2,500 cm^2^ and a depth of 35 cm. Water temperature was maintained at 32–35 °C. At the end of the exercise, mice were carefully towel dried and kept in a drying enclosure at 30 °C for 1 hour. All mice were able to complete this training. This exercise regime was continued until mice were aged 18–20 weeks before euthanising [36] (*n* = 20).

For the exercised obese model, there were two additional control groups. The first group was a diet-induced obesity group, which were high fat fed as described above, and were euthanised after 5 weeks (the time point at which exercised groups commenced training) to examine mouse phenotype and PVAT function at this time point (*n* = 10; Supplementary Figure 1). This control was used to determine that PVAT function is already lost by this time point, resulting in hypertension and hyperglycaemia, confirming that exercise reverses dysfunction as opposed to preventing dysfunction. Another control group of mice were maintained on a standard chow diet and exercised along the same time line as the obese exercised group; five times per week for 60 min (*n* = 8; Supplementary Figure 2). There were no changes in PVAT function or the phenotype of these mice, eliminating the possibility that the stress of water exposure and/or exercise is the cause for any changes in exercised obese mice. For all animal model timelines, see Supplementary Figure 3.

### Blood Plasma Studies

Collected blood samples were centrifuged at 3000×*g* for 10 min at 4 °C to separate plasma. Plasma samples were then stored at − 80 °C until required. Concentration of plasma NA, adiponectin, and insulin was measured using commercially available ELISA kits according to manufacturer’s instructions (Table 1). All samples and standards were measured in duplicate, and the optical density of the zero standard was subtracted from each value. Standard curves were fitted using non-linear regression analysis with a sigmoidal four parameter logistic curve to interpolate values.

**Table 1:** Commercially available ELISA kits used to measure plasma NA, adiponectin, and insulin

Antigen	Name of kit	Supplier	Catalogue no.	Sample dilution
Adiponectin	Adiponectin mouse ELISA kit	Life Technologies Ltd	KMP0041	Plasma—1 in 20,000 PVAT supernatant—1 in 100
Insulin	Insulin mouse ELISA kit	Invitrogen	EMINS	1 in 2
Noradrenaline	NA/NE ELISA kit	Elabscience	E-EL-0047	None

### Wire Myography

Second-order mesenteric arteries were prepared as previously described [37]. Arterial segments (< 250 μm) with PVAT removed or left intact were dissected in ice cold physiological salt solution (PSS, in mmol L^−1^; NaCl, 119; KCl, 4.7; MgSO_4_, 1.17; NaHCO_3_, 25; KH_2_PO_4_, 1.17; EDTA, 0.03; glucose, 5.5; and CaCl_2_, 1.6) and were mounted on 40-μm diameter wires in a wire myograph (Danish MyoTech, Denmark). Arteries were allowed to equilibrate for 30 min at 37 °C in PSS bubbled with 95% air/5% CO_2_ to maintain a pH of 7.4. Following equilibration, vessel starting tension was standardised using a standard normalisation procedure [38]. Vessels were left for a further 30-min equilibration period. To test vessel viability, vessels were challenged with high K^+^ PSS (KPSS, in mmol L^−1^; NaCl, 63.7; KCl, 60; MgSO_4_, 1.17; NaHCO_3_, 25; KH_2_PO_4_, 1.17; K_2_ EDTA, 0.03; glucose, 5.5; and CaCl_2_, 1.6). Vessels were stimulated using an electrical field stimulation protocol over a frequency range of 0.1–30 Hz (20 V, 4 s stimulus duration, 0.2 ms pulse duration) [37]. Stimulation was conducted using a Danish MyoTech CS4 stimulator. LabChart 7 acquisition software (RRID:SCR_001620, AD Instruments, UK) was used to continuously record responses.

#### Pharmacological Assessment

Previously published pharmacological assessment which has been conducted in control vessels were repeated in obese and exercised obese vessels in this study [37]. β_3_-adrenoceptor agonists and antagonists were used to delineate their role in mediating the vascular response to EFS in the presence or absence of PVAT; following the first EFS stimulation, β_3_-adrenoceptor agonist CL-316,243 (10 μM) or β_3_-adrenoceptor antagonist SR59230A (1 μM) was added to ±PVAT vessels and incubated for 30 min, before re-stimulating the vessels. The effects of globular adiponectin (5 μg/ml) were tested on only −PVAT vessels. Vessels were stimulated once and allowed to recover for 15 min before adding adiponectin. The adiponectin was incubated for 5 min before repeating the EFS protocol.

To examine the role of nitric oxide and NA transport, a third arterial preparation was used whereby a section of exogenous PVAT was suspended above a clean artery (Ref no. [37] for illustration of all arterial preparations). This allowed the removal of PVAT for incubation with OCT3 inhibitor corticosterone (100 μM), and eliminated interference with NA uptake within the artery.

Time controls were conducted alongside all experiments to ensure that any change in vascular responsiveness was not a time effect (data not shown).

#### Adiponectin Assay

To quantify adiponectin secretion from obese PVAT compared with healthy controls, PVAT was collected from the mesenteric bed of control and obese mice and weighed. As previously described [4], harvested PVAT was incubated in PSS for 30 min bubbled with 95% air/5% CO_2_ before stimulating using EFS at 10 Hz for 4 s (20 V, pulse duration 0.2 ms). The supernatant was collected and stored at − 80 °C. An adiponectin ELISA kit (Table 1) was used to quantify adiponectin secretion according to manufacturer instructions. Concentration was determined through measurement of absorbance using a plate reader (BioTek, Northstar Scientific, UK) at 450 nm and was calculated per 100 mg of PVAT to account for variation in the amount of PVAT collected from each mesenteric bed.

#### Drug Preparations

CL-316,243, and SR59230A were obtained from Tocris biosciences (R&D systems, UK) and dissolved in PSS. Recombinant mouse globular adiponectin was obtained from Generon (UK, cat no. cyt-432-50μg). Corticosterone (Sigma Aldrich, UK) was dissolved in 100% ethanol. Vehicle controls confirm no effect of 100% ethanol (data not shown).

#### Immunohistochemistry

β_3_-adrenoceptor, OCT3, and tumour necrosis factor alpha (TNFα) expression was assessed in control, obese, and exercised obese PVAT by immunohistochemistry [37]. In brief, sections of PVAT from control, obese, and exercised obese mice embedded in KP-CryoCompound (Klinipath BV, The Netherlands) and 12-μm sections were made using a cryostat. Heat-induced antigen retrieval followed by blocking of exogenous peroxide activity was performed. Sections were blocked with goat serum before incubation with primary antibodies for β_3_-adrenoceptors (10 μg/ml, Abcam, Cambridge, UK, cat no. ab59685, RRID:AB_2225531), OCT3 (10 μg/ml, Sigma Aldrich, UK, cat no. AV44026, RRID:AB_1854770), or TNFα (10 μg/ml, Abcam, Cambridge, UK, cat no. ab6671, RRID:AB_305641) overnight at 4 °C. Slides were then incubated with a biotinylated goat anti-rabbit secondary antibody (2 μg/ml, Abcam, UK, cat no. ab6720, RRID:AB_954902) for 1 hour at room temperature before addition of vectastain avidin-biotin complex (Vector Laboratories, UK) and 3,3′-diaminobenzidine solution (Vector Laboratories, UK) for detection of antibody binding. Images were captured using a colour camera (Leica DFC450, Leica Microsystems, Germany) mounted on a microscope (Leica DM5000, Leica Microsystems, Germany).

Mouse kidney was used as a positive control, and negative controls were conducted using samples incubated with phosphate buffered saline in place of the primary antibody. Controls are provided in Supplementary Figure 4.

#### Statistical Analysis

Data are expressed as the mean ± SEM. All statistical analysis was performed using GraphPad Prism v7 (RRID:SCR_002798, GraphPad Software USA). *P* values < 0.05 were considered statistically significant. Normal distribution was confirmed using the Shapiro-Wilk normality test, supported by Skewness and Kurtosis coefficients (using an acceptable range of ± 2).

#### Phenotype Data

Differences in mouse phenotype measures including blood pressure, blood glucose, organ weights, and plasma ELISA data were analysed using a one-way analysis of variance (ANOVA) followed by a Tukey post hoc test to compare individual groups. For body weight progression, differences were tested using a two-way ANOVA. A minimum sample size of 20 was used for each group. For blood pressure, the average of the 10 measurement cycles was used from each mouse [39].

#### Wire Myography

Contractile responses were normalised to the maximum contraction elicited with KPSS, as consistent with other studies [6, 16, 37, 40] and are expressed as a percentage. Differences between the frequency-response curves of ±PVAT vessels were tested using a two-way ANOVA, with a Bonferroni post-hoc test. Before and after treatments within the same vessel types were compared using a repeated-measures ANOVA, again followed by a Bonferroni post-hoc test. Due to expense, an n of 4 was used for exogenous adiponectin experiments. For all other experiments an n of 8 was used.

#### Immunohistochemistry

Quantitative analysis of immunostaining was performed using ImageJ (RRID:SCR_003070, v1.47, NIH, USA). Images were taken from five fields of view per mouse. The intensities of staining for β_3_-adrenoceptors and OCT3, which were localised to adipocyte membranes, were quantified within/around five arbitrarily selected adipocytes, i.e., staining intensity per adipocyte, as opposed to using a set sized area for staining. This was to account for changes in adipocyte size. TNFα staining was quantified within the whole field of view. Correlation of adipocyte area and intensity of adipocyte membrane staining were assessed to confirm that changes in intensity were not due to changes in adipocyte size (See Supplementary data 5). Statistical differences were analysed using a one-way ANOVA followed by a post hoc Tukey’s test to compare individual groups. Sample sizes of five were used for each group.

## Results

### High Fat Feeding Resulted in Metabolic Syndrome and Autonomic Dysfunction, which Is Reversed Using Exercise

**Fig. 1:**
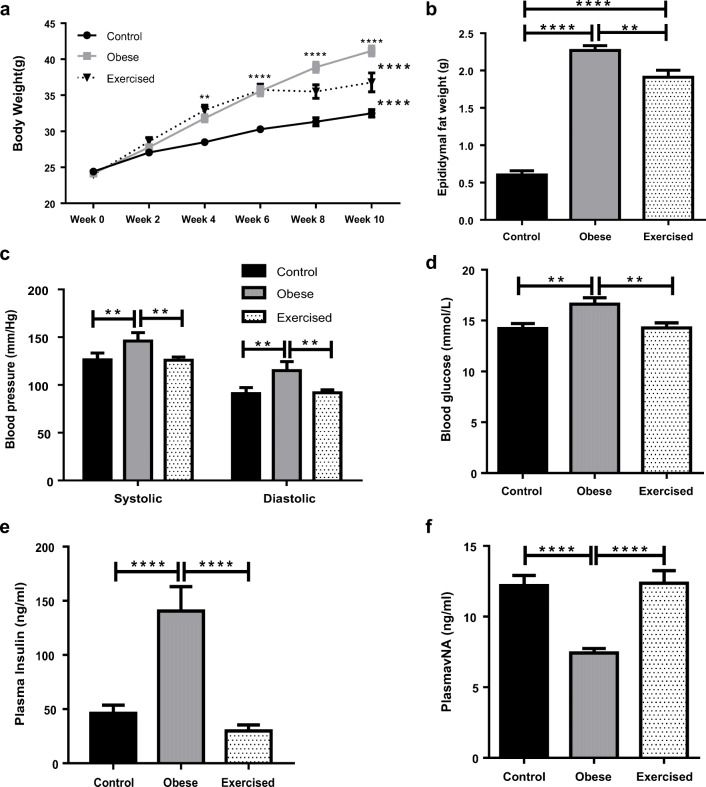
High fat feeding results in obesity and metabolic syndrome which is reversed by exercise. **a** Body weights of control, obese, and exercised obese mice were recorded every 2 weeks (control *n* = 20, obese *n* = 44, exercised *n* = 20). **b** Following sacrifice, epididymal fat pads were removed and weighed (control *n* = 20, obese *n* = 44, exercised *n* = 20). **c** Prior to sacrifice, blood pressure was recorded using a CODA tail cuff system (*n* = 20 all groups). **d** Following sacrifice, blood was collected and glucose concentration was measured using an automatic monitor (control *n* = 20, obese *n* = 44, exercised *n* = 20). Blood was collected and centrifuged to separate plasma. Plasma concentrations of insulin (**e**) and NA (**f**) were measured using ELISA kits (control *n* = 22, obese *n* = 22, exercised *n* = 20). Data shown are mean ± SEM (**P < 0.01, ****P < 0.0001) (NA: noradrenaline)

Obesity was established in C57BL/6j mice by high fat feeding for 10–12 weeks. High fat feeding resulted in a significant increase in body weight in both obese and exercised obese mouse groups (Fig. 1a, control *n* = 20, obese *n* = 44, exercised *n* = 20. Control vs obese *P* < 0.0001, control vs exercised obese *P* < 0.0001). The difference in body weight became significant after 4 weeks of high fat feeding (*P* < 0.01). At week 6, the exercised obese group commenced swimming. This group exhibited no further significant increases in weight gain; however, these mice did not lose any weight. To confirm that changes in weight were due to increases in fat depots and not muscle mass, epididymal fat pads were removed and weighed (Fig. 1b, control *n* = 20, obese *n* = 44, exercised obese *n* = 20). High fat feeding resulted in a significant increase in fat depots in both obese and exercised obese groups (control vs obese *P* < 0.0001, obese vs exercised obese *P* < 0.01, control vs exercised obese *P* < 0.0001).

High fat feeding resulted in significant elevations in both systolic and diastolic blood pressures in the obese group; however, in the exercised obese group, blood pressure was comparable with healthy control mice (Fig. 1c, *n* = 20 all groups. Both systolic and diastolic: control vs obese *P* < 0.01, obese vs exercised obese *P* < 0.01, control vs exercised obese *P* > 0.05). There were no changes in heart or kidneys weights (Supplementary Figure 6) indicating that hypertension cannot be attributed to cardiac hypertrophy or kidney dysfunction. In a similar fashion to blood pressure, high fat feeding resulted in hyperglycaemia in the obese group; however, following exercise, blood glucose concentration normalised (Fig. 1c, control *n* = 20, obese *n* = 44, exercised obese *n* = 20. Control vs obese *P* < 0.01, obese vs exercised obese *P* < 0.01, control vs exercised obese *P* > 0.05).

ELISA kits were used to measure plasma insulin (Fig. 1e) and NA (Fig. 1f) (control and obese groups *n* = 22, exercised obese *n* = 20). Plasma insulin was significantly increased in obesity, which combined with hyperglycaemia indicating possible type II diabetes. Insulin was reduced in the exercised obese group (control vs obese *P* < 0.0001, obese vs exercised obese *P* < 0.0001, control vs exercised obese *P* > 0.05), and plasma NA was significantly reduced in obesity, indicating autonomic dysfunction. In exercise, NA was restored to control levels (control vs obese *P* < 0.0001, obese vs exercised obese *P* < 0.0001, control vs exercised obese *P* > 0.05).

**Fig. 2:**
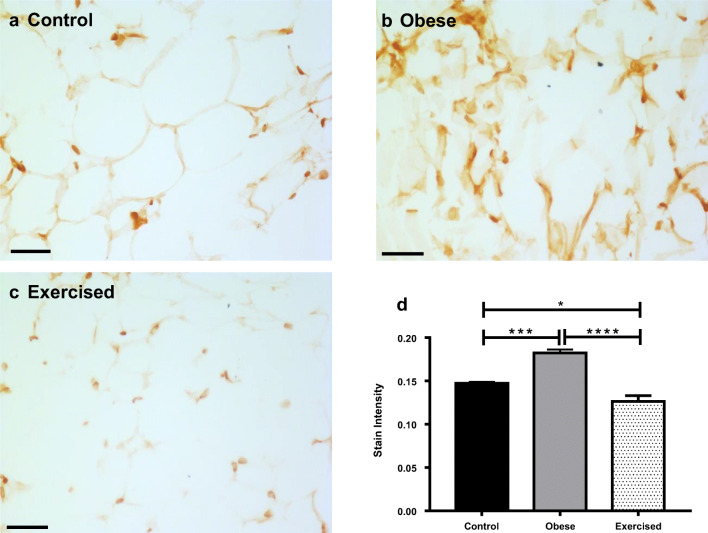
Exercise reduced inflammation in obesity. Sections of mouse mesenteric PVAT from control mice (**a**), obese (**b**), and exercised obese mice (**c**) were stained for TNFα. Scale bar represents 50 μm. Staining intensity in the whole field of view was quantified (**d**). Data shown are mean ± SEM. Differences were tested using a one-way ANOVA (*n* = 5 both groups, *P < 0.05, **P < 0.01, ****P < 0.0001) (TNFα: tumour necrosis factor-α)

Expression of the inflammatory cytokine TNFα was examined using immunohistochemistry in sections of mesenteric PVAT from control, obese, and exercised obese mice (Fig. 2; *n* = 5 all groups). In obese mice, staining intensity of TNFα in PVAT was significantly increased compared with controls (*P* < 0.001). In obese exercised mice, expression of TNFα was significantly reduced as compared with both obese (*P* < 0.0001) and control mice (*P* < 0.05) (for positive and negative controls, see Supplementary Figure 4), suggesting that exercise has substantial beneficial effects alleviating inflammation in obesity.

### Exercise Successfully Restored PVAT Function in Obesity

**Fig. 3:**
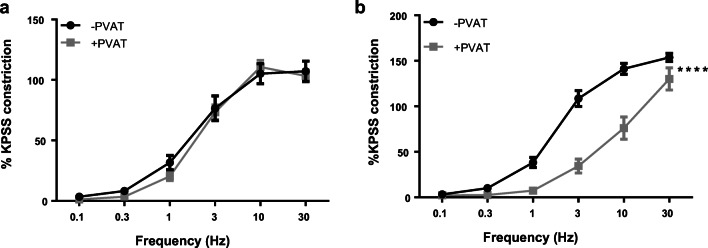
PVAT anti-contractile dysfunction in obesity is recused with exercise. Vessels with and without PVAT from obese (**a**) and exercised obese (**b**) mice were subjected to EFS. Data shown are mean ± SEM (*n* = 8 for both, ****P < 0.0001) (EFS: electrical field stimulation; PVAT: perivascular adipose tissue)

To examine the effects of obesity on sympathetic nerve function in PVAT, EFS profiles were generated in mesenteric resistance arteries from obese mice (0.1–30 Hz, 20 V, 0.2 ms pulse duration, 4 s train duration). In obese mice, there was no significant difference between −PVAT and +PVAT vessels (Fig. 3a; *P* > 0.05, *n* = 8). Therefore, electrical activation of sympathetic nerves in obese PVAT no longer elicits an anti-contractile effect. However, electrical activation of sympathetic nerves in exercised obese PVAT elicited a significant anti-contractile effect (Fig. 3b; *P* < 0.0001, *n* = 8), indicating that exercise has restored PVAT function.

### β_3_-Adrenoceptor Expression Is Downregulated in Obesity but Improved with Exercised

**Fig. 4:**
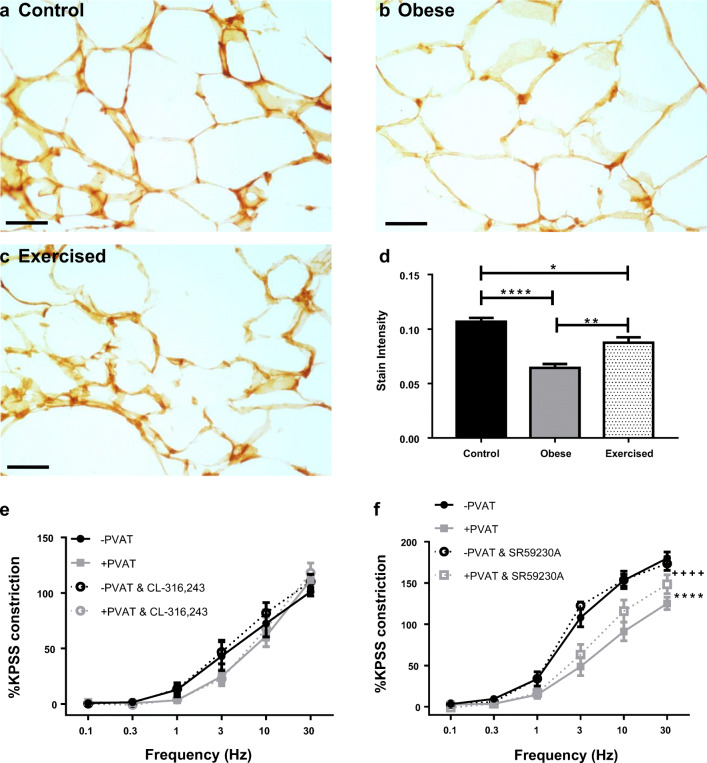
Exercise in obesity upregulates β_3_-adrenoceptor expression to restore anti-contractile function. Sections of mouse mesenteric PVAT from control mice (**a**), obese mice (**b**), and exercised obese mice (**c**) were stained for β_3_-adrenoceptors. Scale bar represents 50 μm. Staining intensity of adipocyte membranes was quantified (**d,** *n* = 5 all groups, *P < 0.05, **P < 0.01, ****P < 0.0001). Following control responses to EFS ±PVAT vessels, obese vessels were incubated for 30 min with CL-316,243 (**e**, *n* = 8, *P* > 0.05), and exercised obese vessels were incubated with SR59230A (**d**, *n* = 8, −PVAT vs +PVAT *P* < 0.0001****, +PVAT vs +PVAT and SR59230A *P* < 0.0001****, −PVAT vs −PVAT and SR59230A *P* > 0.05, −PVAT vs +PVAT and SR59230A *P* < 0.0001****). Data shown are mean ± SEM (PVAT: perivascular adipose tissue)

In a previous study, we have indicated that PVAT function is mediated in part by β_3_-adrenoceptors [37]; therefore, in an attempt to rescue PVAT dysfunction in obesity, the effects of the β_3_-adrenoceptor agonist were tested. Following control stimulations with EFS, ±PVAT vessels from obese mice were incubated for 30 min with CL-316,243, before repeating the stimulus (Fig. 4e). CL-316,243 had no effect on either −PVAT or +PVAT vessels (*P* > 0.05, *n* = 8), indicating that these receptors may be downregulated in obesity.

The expression of β_3_-adrenoceptors was examined using immunohistochemistry in sections of mesenteric PVAT from control, obese, and exercised obese mice (Fig. 4a–d; *n* = 5 all groups). The intensity of staining in the adipocyte membrane was quantified, and in obese mice, expression of the adrenoceptor was significantly reduced as compared with that in obese mice (*P* < 0.0001). In exercised obese mice, expression of β_3_-adrenoceptors is significantly increased compared with obese mice (*P* < 0.01); however, expression is still significantly lower in exercised obese mice than in control mice (*P* < 0.05) (for positive and negative controls, see Supplementary Figure 4).

To determine if the increase in expression of β_3_-adrenoceptors in exercise is sufficient to restore function, following the first EFS stimulation, ±PVAT vessels from exercised obese mice were incubated with β_3_-adrenoceptor antagonist SR59230A (Fig. 4f; *n* = 8). Similar to previously reported data in control mice [37], SR59230A resulted in a significant reduction in the PVAT anti-contractile effect (−PVAT vs +PVAT *P* < 0.0001, −PVAT vs −PVAT and SR59230A *P* > 0.05, +PVAT vs +PVAT and SR59230A *P* < 0.0001, −PVAT vs +PVAT and SR59230A *P* < 0.0001). These results indicate that exercise restored β_3_-adrenoceptor-mediated PVAT function in obesity.

### OCT3 Expression Is Downregulated in Obesity and Is Reversed with Exercise

**Fig. 5:**
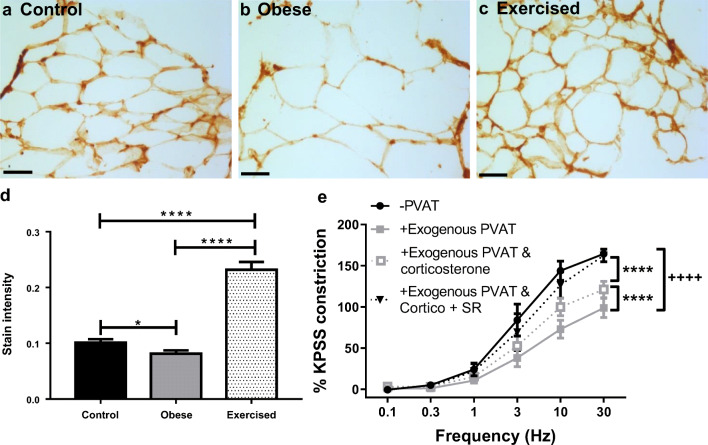
Exercise upregulates OCT3, restoring NA uptake into adipocytes. Sections of mouse mesenteric PVAT from control mice (**a**), obese mice (**b**), and exercised obese mice (**c**) were stained for OCT3. Scale bar represents 50 μm. Staining intensity of adipocyte membranes was quantified (**d**, *n* = 5 all groups, *P < 0.05, ****P < 0.0001). **e** Following EFS, exercised obese vessels were incubated with corticosterone alone for 30 min and subjected to a second EFS protocol. Following this, vessels were incubated for a second time with corticosterone plus SR59230A (SR) for 30 min, before being stimulated with EFS for a final time (*n* = 8, −PVAT vs +exogenous PVAT *P* < 0.0001^++++^, +exogenous PVAT vs +exogenous PVAT and corticosterone *P* < 0.0001****, −PVAT vs +exogenous PVAT and corticosterone *P* < 0.0001****, +exogenous PVAT vs +exogenous PVAT and corticosterone + SR *P* < 0.0001****, −PVAT vs +exogenous PVAT and corticosterone + SR *P* > 0.05). Data shown are mean ± SEM (EFS: electrical field stimulation; NA: noradrenaline; OCT3: organic cation transporter 3)

We have previously demonstrated that the PVAT anti-contractile effect in health is mediated by a combination of β_3_-adrenoceptor-mediated anti-contractile factor release, and NA uptake into adipocytes by OCT3 [37]. Therefore, using immunohistochemistry, we examined the expression of OCT3 in control, obese, and exercised obese PVAT (*n* = 5 all groups; Fig. 5a–d). The intensity of staining in the adipocyte membrane was quantified, and in obese mice, expression of OCT3 was significantly decreased compared with control mice (*P* < 0.05) and control mice (*P* < 0.0001, *n* = 5 for all groups). However, in the exercised obese PVAT, OCT3 expression was significantly increased compared with both control and obese groups (control vs exercised obese *P* < 0.0001, obese vs exercised obese *P* < 0.0001) (for positive and negative controls, see Supplementary Figure 4).

PVAT denuded vessels with sections of exogenous PVAT suspended above were subjected to control EFS stimulations, before removing the exogenous PVAT and incubating with the OCT3 inhibitor corticosterone (Fig. 5e). Following 30-min incubation, the exogenous PVAT was re-suspended above the vessel, and the EFS protocol was repeated. Similar to previously published data in control mice [37], corticosterone induced a partial inhibition of the anti-contractile effect (*P* < 0.0001, *n* = 8). Next, the exogenous PVAT was removed again and incubated with both corticosterone and SR59230A, and the restored anti-contractile effect in exercised obese mice was completely abolished (*P* < 0.0001, *n* = 8), suggesting that the normal functioning of both β_3_-adrenoceptors and OCT3 had been restored by exercise.

### Adiponectin Is a Vasodilator in Exercised Obese Arteries But Not in Obese Arteries

**Fig. 6:**
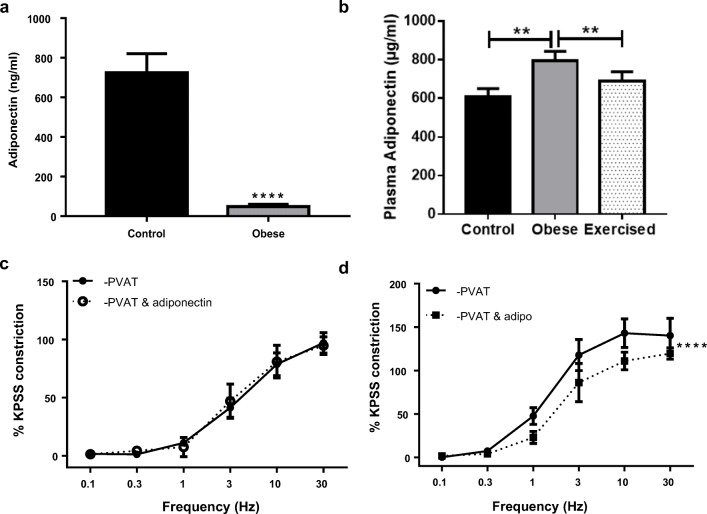
Adiponectin secretion in obesity is reduced and its vasodilator function is lost. **a** Mesenteric PVAT from control and obese mice was collected and adiponectin secretion per 100 mg was measured (*n* = 12 both groups, *P* < 0.0001****). Following sacrifice, blood was collected and centrifuged to separate plasma. Plasma concentrations of adiponectin (**b**) were measured using an ELISA kit (control *n* = 22, obese *n* = 22, exercised *n* = 20, *P* < 0.01**). Following the control EFS protocol, PVAT-denuded vessels from obese (**c**) and exercised obese (**d**) were allowed 15 min to recover before incubating with recombinant globular mouse adiponectin for 15 min and repeating the stimulus (*n* = 4 for both, *P* < 0.0001****). Data shown are mean ± SEM (EFS: electrical field stimulation; PVAT: perivascular adipose tissue)

Previously, we have reported that adiponectin is the anti-contractile factor released upon β_3_-adrenoceptor activation [37]; therefore, we measured the secretion of adiponectin from obese PVAT compared with secretion from healthy control PVAT. PVAT was collected from the mesenteric beds of control and obese mice and stimulated at 10 Hz for 4 s. The surrounding supernatant was collected and adiponectin concentration was quantified using an ELISA. The concentration of adiponectin secreted from the obese PVAT was significantly reduced as compared with the healthy control (Fig. 6a; *n* = 12 both groups, *P* < 0.0001).

An ELISA kit was used to measure plasma adiponectin (Fig. 6b) (control and obese groups *n* = 22, exercised obese *n* = 20). Plasma adiponectin was significantly increased in obesity and was reduced back to control levels with exercise (control vs obese *P* < 0.01, obese vs exercised obese *P* < 0.01, control vs exercised obese *P* > 0.05).

To determine if adiponectin presents a viable target for treating the vascular complications of obesity, the effects of globular adiponectin were tested in obese and exercised obese mice. Following control stimulations with EFS, −PVAT vessels were given 15 min to recover before incubating with exogenous adiponectin for a further 5 min. In obese vessels, exogenous application of adiponectin no longer elicits a vasodilator effect (Fig. 6c; *P* > 0.05, *n* = 4). However, in exercised obese mice, adiponectin did cause a vasodilation (Fig. 6d; *P* < 0.0001, *n* = 4), indicating that the impaired vasodilator function of adiponectin in obesity has been restored by exercise.

## Discussion

This study has for the first time investigated the effects of autonomic dysfunction on the PVAT effect in obesity and the potential for exercise in restoring PVAT function in resistance arteries. The main findings of this study were (1) in obesity, PVAT becomes inflamed, and this inflammation is reversed with exercise; (2) the sympathetic nerve-mediated PVAT anti-contractile effect is lost in obesity, but can be rescued using exercise; (3) reversal of PVAT dysfunction in obesity using exercise is accompanied by normalised blood pressure, blood glucose, and plasma insulin, independent of weight loss; (4) expression of β_3_-adrenoceptors and OCT3 are downregulated in obesity, and activation of β_3_-adrenoceptors cannot restore function; (5) adiponectin secretion from mesenteric PVAT is reduced in obesity; and (6) the vasodilator function of adiponectin is lost in obesity but can be rescued using exercise.

We found that 10–12 weeks of high fat feeding induced increased adiposity, hypertension, and hyperglycaemia. Plasma insulin was also increased threefold, which, in combination with hyperglycaemia, would suggest that these mice may have type II diabetes, although glucose tolerance testing is required to confirm. In humans, metabolic syndrome is defined by a combination of at least three out of five conditions: abdominal obesity, hypertension, type-II diabetes, hypertriglyceridemia, and hypercholesterolemia [41]. Therefore, these results are indicative of metabolic syndrome. Consistent with other exercise studies, we have demonstrated that exercise relieves the vascular and metabolic complications of obesity, i.e., hypertension, hyperglycaemia, and hyperinsulinemia [42–44]. These findings were independent of weight loss, which fits with the “fat but fit” hypothesis, proposing that cardiorespiratory fitness may be a more important determinant of mortality than weight [45]. Hypertension and type II diabetes are major risk factors in CVD and contribute to the short life expectancy of obese patients; therefore, treatment of these vascular diseases using this exercise model may extend the life expectancy of obese patients.

We reported a reduced level of circulating NA in our obese mouse model, and a similar reduction in plasma NA in obesity has been reported in human patients [46]. However, measurements of plasma NA and urinary metabolites are only indicative of autonomic dysfunction and not nerve activity, as these measurements do not take into account dysfunction that may be occurring downstream, e.g., NA uptake into the periphery [47]. Therefore, reduced circulating NA does not mean reduced nerve activity and only confirms dysfunction [46]. In obesity, it is widely accepted that sympathetic nerves become overactive in both rodents and humans [8, 9, 48]. Indirect recordings such as plasma NA or responses to autonomic blockade are often used for a rough guide on changes in autonomic function; however, direct recordings of sympathetic nerve activity would provide a more accurate investigation of autonomic over-activity in our model of obesity [49]. However, currently, there are no commercially available telemeter devices to measure sympathetic nerve activity in conscious mice. Direct recordings can be taken in anaesthetised mice, but these preparations raise concerns regarding the effects of anaesthetic on sympathetic nerve activity and arterial blood pressure, which is intimately linked to sympathetic nerve activity and is greatly reduced under anaesthesia [50]. There has been some success by Hamza and Hall [51] in developing an experimental method for direct recordings of renal sympathetic nerve activity in conscious mice; however, the viability of nerve recordings decreased rapidly in the days following surgery, making this method unsuitable in its current state for recording nerve activity before and after high fat feeding.

Activation of β_3_-adrenoceptors using an agonist did not rescue loss of function, indicating either dysfunction or downregulation of the receptor. There are no agonists available for OCT3; therefore, we were unable to determine if pharmacological manipulation of this transporter could restore function. In this study, we have indicated a downregulation of both β_3_-adrenoceptors and OCT3 using immunohistochemistry, which is not a quantitative measure and is therefore a limitation of this study. In heart failure, over-activity of sympathetic nerves results in desensitisation and internalisation of β_3_-adrenoceptors [10]. In future studies, we aim to demonstrate using western blots and quantitative PCR that a similar process is occurring in obese adipose tissue, resulting in a reduction in expression of β_3_-adrenoceptors and OCT3. It has been suggested that β_3_-adrenoceptors are a potential target for the treatment of hypertension in obesity, as agonists have been shown to be hypotensive in healthy canines and rodents [52, 53]; however, reduced expressions of β_3_-adrenoceptors in obesity make the application of an agonist redundant. Reduced expression of β_3_-adrenoceptors in obesity has been shown in a number of obese mouse models [54]; however, this is the first study to report a change in expression of OCT3 in obesity.

The reduced expression of β_3_-adrenoceptors in obesity results in a reduction in adiponectin secretion as demonstrated in this study using an ELISA. Reduced secretion of the vasodilator adiponectin will contribute to increases in arterial tone and blood pressure. Paradoxically, we found plasma adiponectin levels to be elevated in our obese mice. Studies have shown that adiponectin clearance in obesity is impaired [57]; therefore, it is possible that this has resulted in an accumulation of adiponectin in the plasma of our mice. Typically, plasma adiponectin is accepted as reduced in obesity [11, 55, 56]. The obese model used here is a relatively acute model of obesity. It is possible that this accumulation of adiponectin is an early compensatory mechanism in obesity in response to reduced secretion, and levels will decline with chronic obesity. Nonetheless, with exercise, plasma adiponectin levels were returned to normal, indicating a restorative effect of exercise on adiponectin secretion and clearance.

To determine if administration of adiponectin would be useful in obesity, we applied exogenous adiponectin to obese arteries. However, the vasodilator effects of adiponectin were absent. This may suggest that increased plasma adiponectin may have resulted in desensitisation of receptors. In addition, adiponectin causes vasodilation via mechanisms dependent upon adenosine monophosphate-activated protein kinase (AMPK) [58, 59]. In obesity, phosphorylation of AMPK is reduced; supressing AMPK activity [60, 61]. It is possible that the lack of adiponectin function in obesity may be due to changes in AMPK activity in the vascular smooth muscle and endothelial cells. However, in this study, we were able to restore the vasodilator effects of adiponectin in obesity using exercise.

This is the first study to demonstrate a restoration of the PVAT anti-contractile effect in resistance arteries in obesity using exercise. Moreover, these findings were independent of weight loss. One of the beneficial effects in PVAT of exercise is reduced inflammation. There is a clear reduction in the expression of TNFα, an important inflammatory marker [22] in exercised obese PVAT. This will therefore reduce the damage caused to PVAT by inflammation. The results of the TNFα expression studies are consistent with previous studies in our laboratory, whereby bariatric surgery in obese patients restored anti-contractile function in PVAT and reduced inflammation in PVAT, whilst patients were still in the obese body mass index (BMI) range [32].

We are the first to demonstrate that expression of β_3_-adrenoceptors and OCT3 are increased by exercise. Expression levels of OCT3 have been returned to control levels, whereas expression of β_3_-adrenoceptors is still below normal. However, the efficacy of the β_3_-adrenoceptor antagonist in reducing the anti-contractile effect alone, and abolishing the anti-contractile effect in combination with the OCT3 inhibitor would suggest that the expression of β_3_-adrenoceptors has increased enough to restore the healthy PVAT anti-contractile mechanism. Therefore, healthy sympathetic activity, i.e., exercise, has restored PVAT’s ability to release an anti-contractile factor upon β_3_-adrenoceptor activation, and the ability to transport NA into adipocytes via OCT3, preventing the NA from reaching the blood vessel and eliciting contraction.

Many studies have indicated that exercise can induce “beiging” of white adipose tissue [62–65]. This process, by which white adipocytes can be stimulated to differentiate into an intermediate between white and brown adipocyte, results in “beige” or “brite” adipocytes which can participate in thermogenesis, making it metabolically beneficial [63, 66]. The degree of beiging is dependent on the duration and type of exercise [67, 68], and it is possible that this process has been induced in the exercise model used here. Furthermore, both β_3_-adrenoceptors [69–72] and OCT3 [73] have been indicated in beiging. In future studies, we will aim to investigate the potential role of beiging in the beneficial effects of our exercise model.

Dysfunction of the sympathetic nervous system in obese PVAT results in a loss of the PVAT anti-contractile effect, which may be contributing to the development of hypertension and type II diabetes. The over-activity of sympathetic nerves in obesity causes downregulation of β_3_-adrenoceptors and OCT3, resulting in a loss of PVAT function. Moreover, obese arteries are no longer responsive to adiponectin. Healthy sympathetic activity, i.e., exercise, restored the normal PVAT anti-contractile mechanism in obesity, independent of weight loss. Exercise reduced inflammation of PVAT and increased expression of β_3_-adrenoceptors and OCT3 to restore function. Moreover, the vasodilator function of adiponectin was restored. As a result of repaired PVAT function, the vascular complications of obesity, hypertension, and type II diabetes were reversed, indicating exercise as an effective treatment for obesity-related illnesses, highlighting the importance of exercise as a first line of treatment in the vascular complications of obesity.

## Supplementary Information

ESM 1 (PDF 352 kb)

## Abbreviations

EFS: Electrical field stimulation
KPSS: High potassium physiological salt solution
L-NMMA: L-N^G^-monomethyl arginine citrate
NA: Noradrenaline
NOS: Nitric oxide synthase
OCT: Organic cation transporter
PSS: Physiological salt solution
PVAT: Perivascular adipose tissue
TNFα: Tumour necrosis factor alpha
