# Supplementary material for: Restoring Perivascular Adipose Tissue Function in Obesity Using Exercise
Source: Cardiovasc Drugs Ther. 2021 Mar 9;35(6):1291–304. doi: 10.1007/s10557-020-07136-0 (PMC8578065; doi:10.1007/s10557-020-07136-0)
Supplement: Supplementary file 1 — (PDF 352 kb) [file 10557_2020_7136_MOESM1_ESM.pdf]

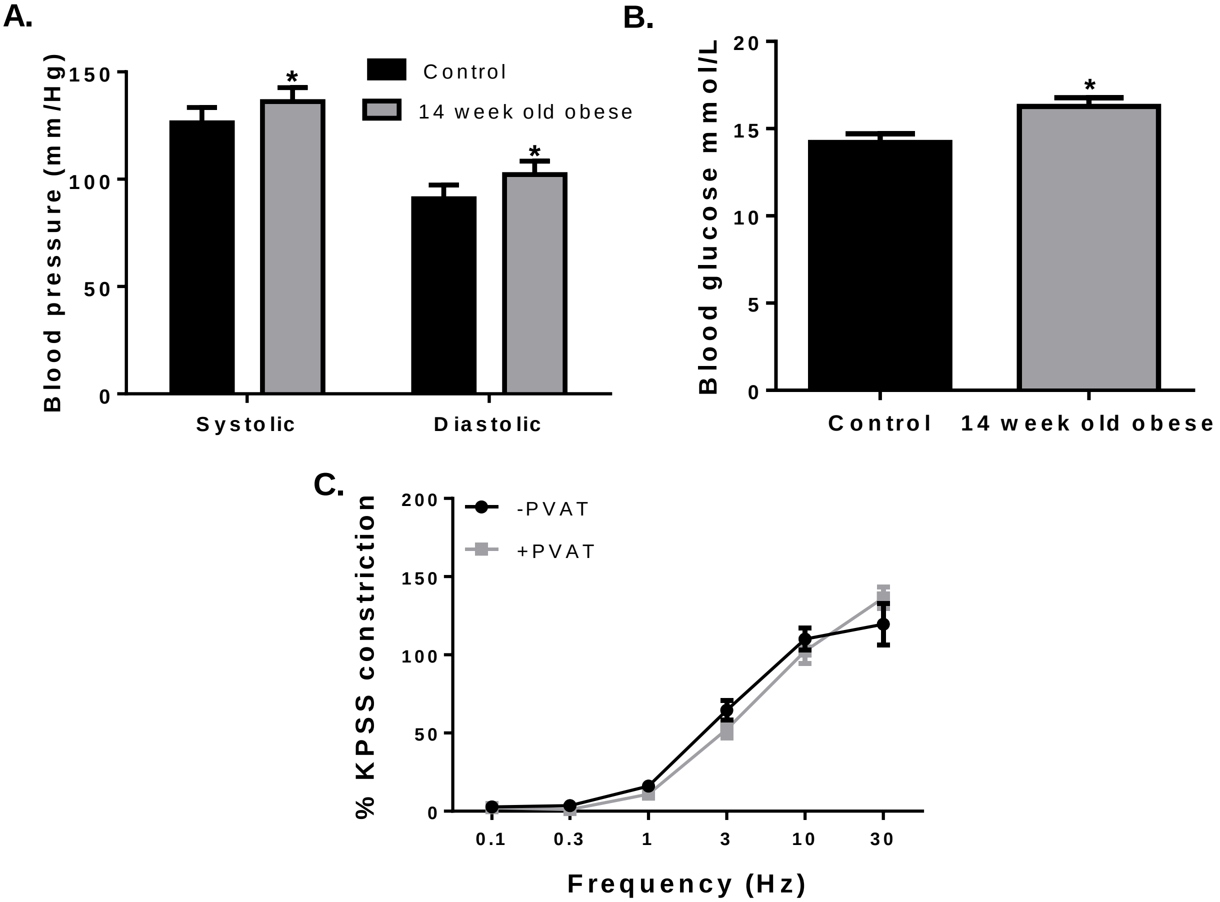

Supplementary Figure 1: The anti-contractile effect is lost after 6 weeks of high fat feeding resulting in hypertension and hyperglycaemia. To determine if exercise reverses or prevents loss of PVAT function, mice fed the high fat diet for 6 weeks until 14 weeks old (the time point at which the exercised group commence swimming) were sacrificed. **A:** Prior to sacrifice blood pressure was measured using a CODA tail cuff system (control n=20, 14 week old obese n=10). **B:** In fasted mice, following sacrifice mixed blood was collected and blood glucose was immediately measured using an automatic monitor (control n=20, 14 week old HFD n=10). High fat feeding for 6 weeks resulted in hypertension and hyperglycaemia. Data shown are mean ± SEM. Differences between groups were tested using an unpaired t-test (*P*<0.05*). **C:** EFS profiles were generated in ±PVAT mesenteric resistance arteries from 14 week old obese mice, and the anti-contractile effect is already lost. Data shown are mean ± SEM, and are expressed as % of the maximum constriction elicited with KPSS. Differences between –PVAT and +PVAT vessels were tested using a two-way ANOVA, followed by a Bonferroni post-hoc test (n=10, *P*>0.05).

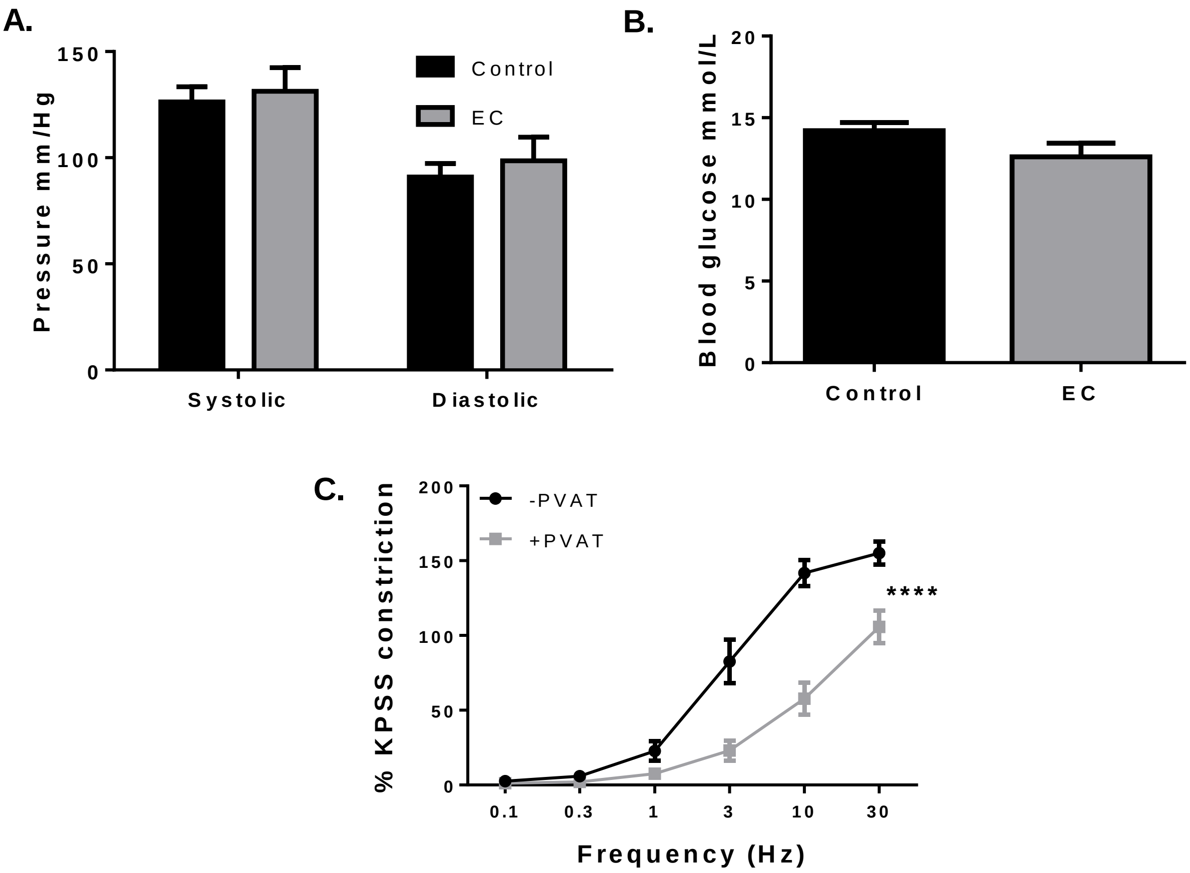

Supplementary Figure 2: Exercise in normal mice has no effect on PVAT function, blood pressure, or blood glucose. To ensure that changes in the exercised obese mice are not due to the stress of exercise or water exposure, a group of control mice fed a normal chow diet (exercised control; EC) were exercised alongside obese mice. **A:** Prior to sacrifice blood pressure was measured using a CODA tail cuff system (control n=20, EC n=8). **B:** In fasted mice, following sacrifice mixed blood was collected and blood glucose was immediately measured using an automatic monitor (control n=20, EC =10). Data shown are mean ± SEM. Differences between groups were tested using an unpaired t-test ($P>0.05$). **C:** EFS profiles were generated in ±PVAT mesenteric resistance arteries from EC mice, and the anti-contractile effect is unaltered. Data shown are mean ± SEM, and are expressed as % of the maximum constriction elicited with KPSS. Differences between –PVAT and +PVAT vessels were tested using a two-way ANOVA, followed by a Bonferroni post-hoc test (n=8, $P<0.0001$****).

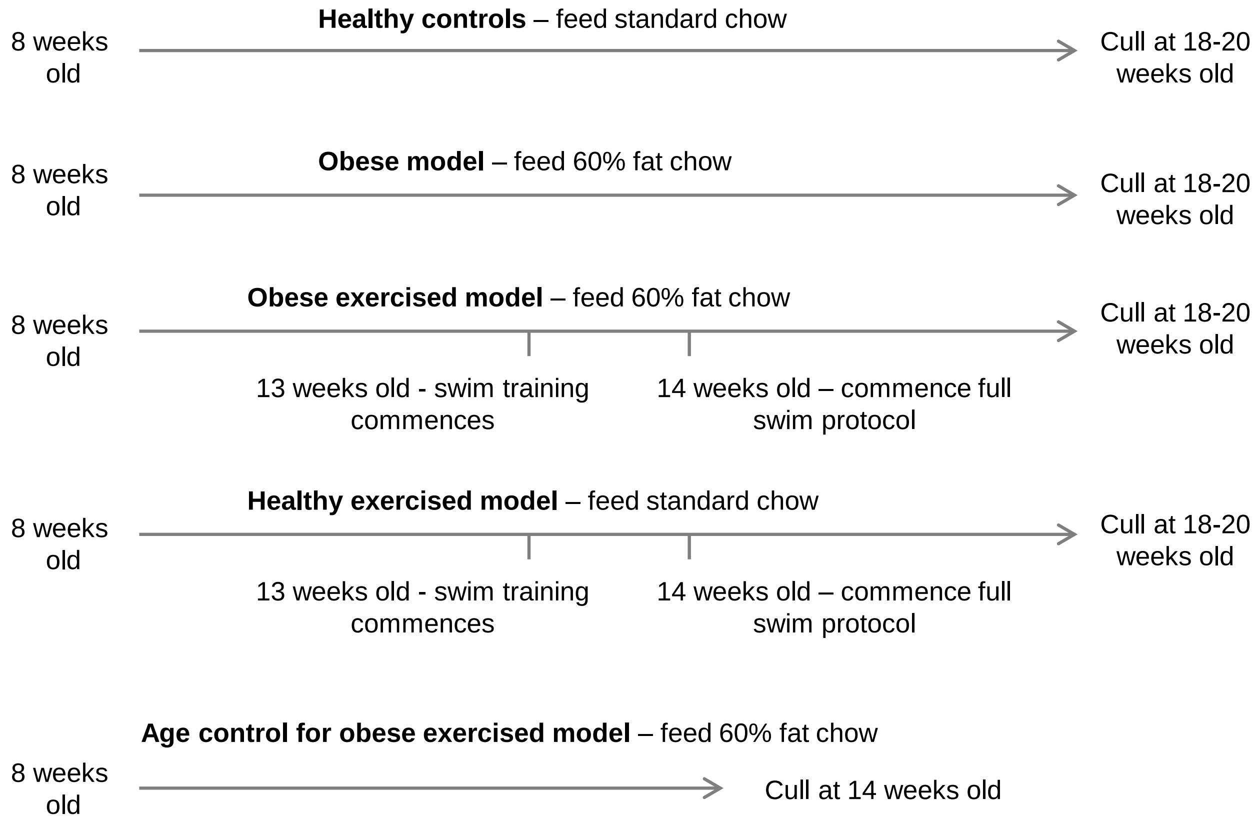

Supplementary Figure 3: Mouse model timelines. Control male C57BL/6j mice were fed a standard chow diet until euthanasia at 18-20 weeks old. For the obese model, mice were fed a 60% fat chow from 8 weeks old until euthanasia. The obese exercised control was fed the high fat diet from 8 weeks old, and swim training commenced at 13 weeks old for 1 week, before starting the full swimming protocol at 14 weeks old until euthanasia. A healthy exercised control group was established. Similar to the obese exercised group, swim training began at 13 weeks old, and the full swim protocol began at 14 weeks old. These mice were maintained on a standard chow diet. Finally, to investigate vessel function in obese mice before the swim protocol begins, an aged-matched group of obese mice was established by feeding C57BL/6j mice a high fat diet from 8 weeks old, until euthanasia at 14 weeks old.

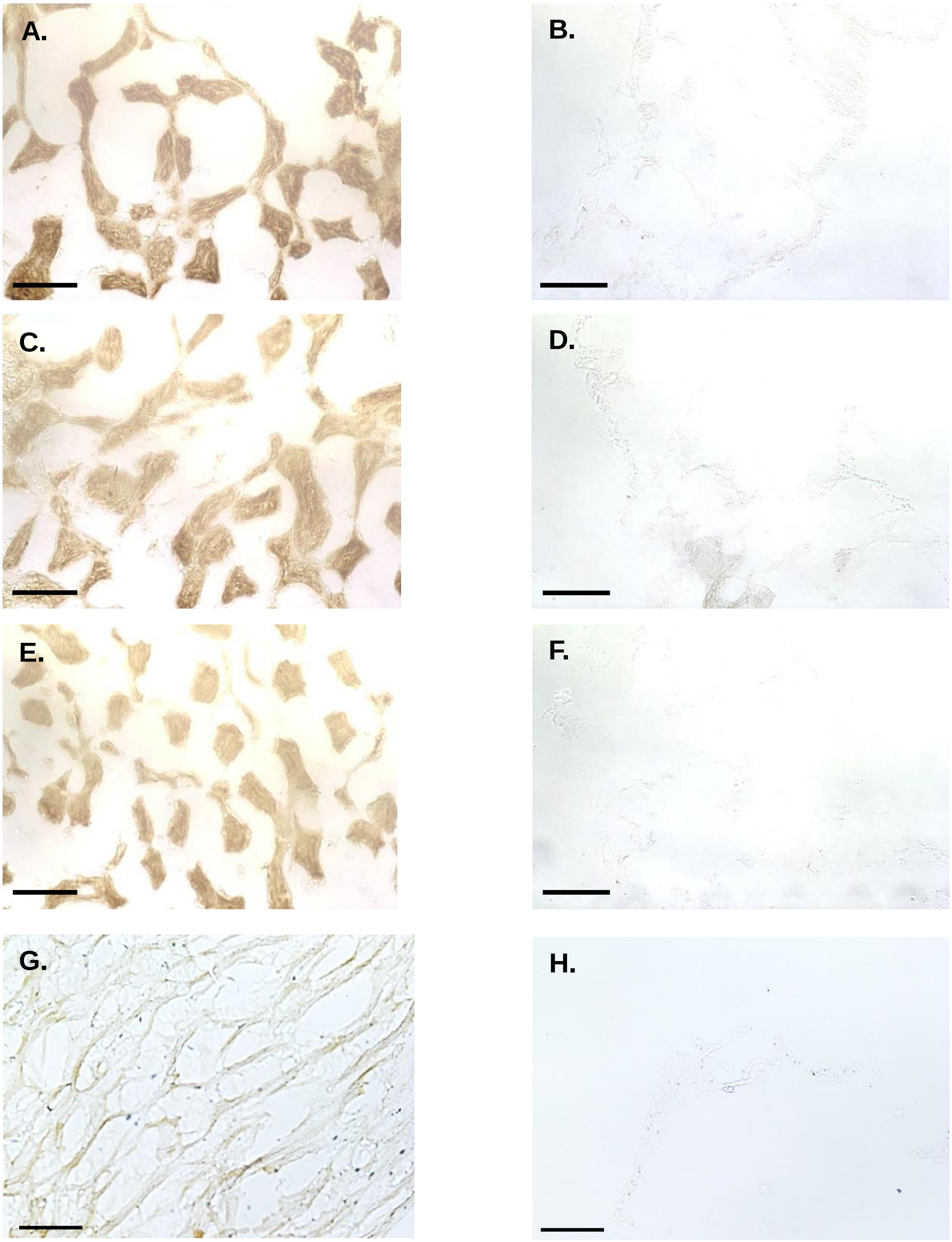

Supplementary figure 4: Positive and negative controls. Positive controls were conducted in kidney for β_3-adrenoceptors (**A**), OCT3 (**C**), TNFα (**E**) and eNOS (**G**). Negative controls whereby sections were incubated with phosphate buffered saline in place of primary antibody for β_3-adrenoceptors (**B**), OCT3 (**D**),TNFα (**F**) and eNOS (**H**) Scale bar represents 50µm.

A.

B.

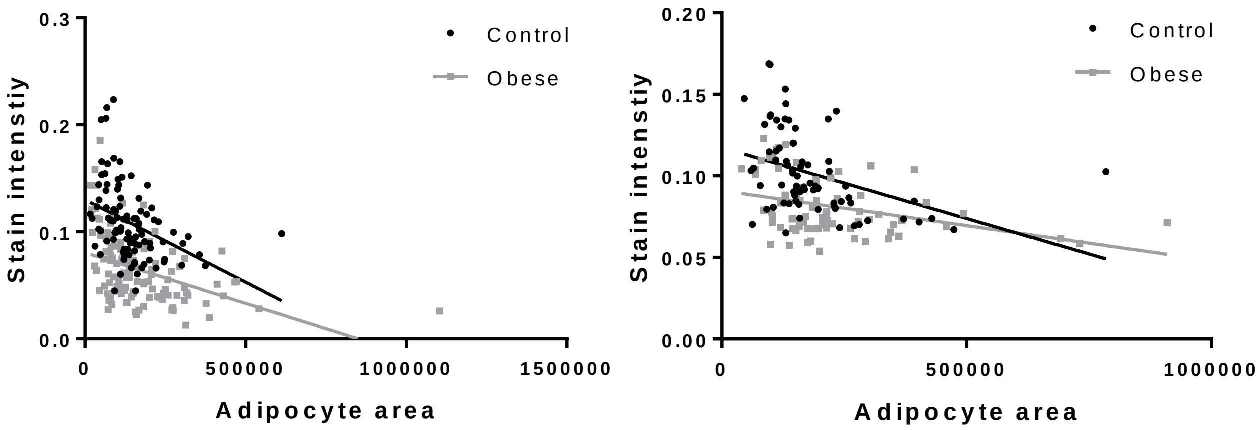

Supplementary figure 5: Stain intensity and adipocyte area correlation. To ensure that the changes in expression of β_3-adrenoceptors (**A**), and OCT3 (**B**) at the adipocyte membrane are not simply due to increased area of the adipocyte, the stain intensity and adipocyte area was correlated. As can be seen in the graph, stain intensity was reduced in obesity in adipocytes with similar areas to controls.

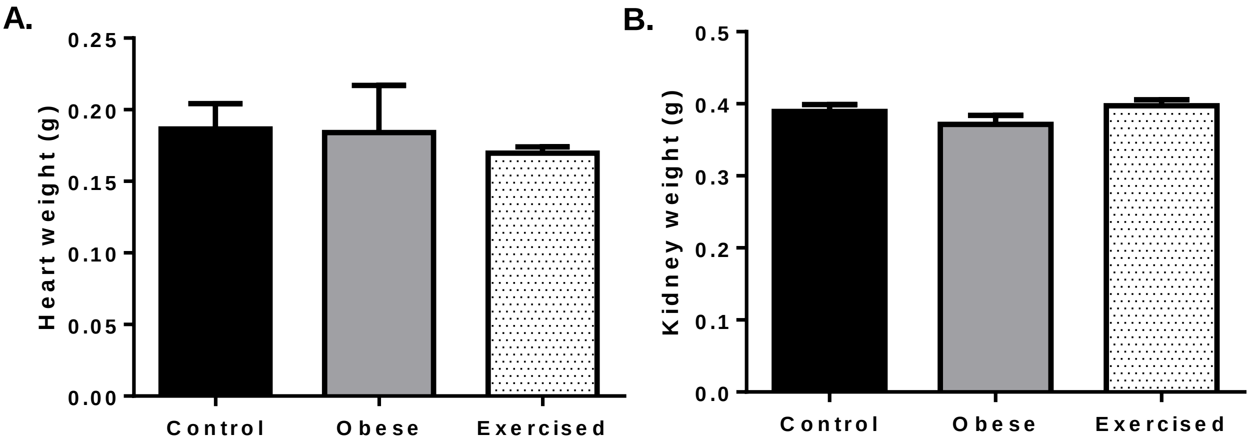

Supplementary figure 6: Organ weights. Following sacrifice, the heart (**A**) and kidneys (**B**), were removed for weighing. Data shown are mean ± SEM. Differences between groups were tested using a one-way ANOVA (control n=20, obese=44, exercised obese n=20).
